# Phytosynthesized Silver Nanoparticles from Waste *Kigelia africana* Flowers: Characterization and Functional Applications

**DOI:** 10.1002/open.70187

**Published:** 2026-03-26

**Authors:** Lakshya, Amanpreet Kaur, Utkarsh Tyagi, Man Vir Singh, Aaysha Pandey, Kamal Kishore, Ranjeet Brajpuria, Soniya Dhiman, Naresh Kumar Wagri

**Affiliations:** ^1^ School of Biotechnology IFTM University Moradabad India; ^2^ Department of Chemistry School of Sciences IFTM University Moradabad India; ^3^ Department of Chemistry Graphic Era (Deemed to be University) Dehradun India; ^4^ School of Allied Sciences, Department of Chemistry Dev Bhoomi Uttarakhand University Dehradun India; ^5^ School of Engineering University of Wollongong Wollongong Australia; ^6^ Applied Science Cluster UPES Dehradun India; ^7^ Helmholtz‐Zentrum Dresden‐Rossendorf Helmholtz Institute Freiberg for Resource Technology Dresden Germany; ^8^ Department of Materials Science and Engineering School of Industrial Engineering and Management KTH Royal Institute of Technology Stockholm Stockholm Sweden

**Keywords:** AgNPs, antidiabetic, antimicrobial, antioxidant, *Kigelia africana*

## Abstract

Plant extracts provide a rapid, cost‐effective, and sustainable route for synthesizing metallic nanoparticles, and various extracts have been used to produce silver nanoparticles. The ethanolic extract of waste *Kigelia africana* flowers exhibited both reduction and stabilization effects. The formation of silver nanoparticles (Ka‐AgNPs) was confirmed by visual color change and further validated using spectroscopic and microscopic techniques. Ultraviolet–Visible spectroscopy of the synthesized nanoparticles showed a characteristic absorption peak at 421.36 nm. Fourier Transform Infrared Spectroscopy (FT‐IR) revealed absorption bands corresponding to phytoconstituents acting as capping agents. Field Emission Scanning Electron Microscopy (FESEM) provided insights into the morphology, while X‐ray Diffraction (XRD) indicated an average crystalline size of 41.56 nm. Antioxidant activity, assessed via 2,2‐Diphenyl‐1‐picrylhydrazyl (DPPH) and 2,2′‐Azino‐bis(3‐ethylbenzothiazoline‐6‐sulfonic acid) (ABTS) radical scavenging assays, yielded IC_50_ values of 27.88 and 17.18μg/mL, respectively. The nanoparticles also exhibited significant antimicrobial activity. Moreover, *α*‐amylase and *α*‐glucosidase inhibition studies demonstrated promising antidiabetic potential, with IC_50_ values of 98.26μg/mL and 125.34μg/mL, respectively. Overall, this study highlights the multifunctionality of silver nanoparticles synthesized using waste K. africana flowers, underscoring their potential medicinal applications.

## Graphical abstract

Green synthesized silver nanoparticles from Kigelia africana flower waste were characterized using Ultraviolet–Visible Spectroscopy (UV–Vis), Fourier Transform Infrared Spectroscopy (FT‐IR), Field Emission Scanning Electron Microscopy (FESEM), and X‐ray Diffraction (XRD). Functional applications were evaluated through antioxidant assays including 2,2‐Diphenyl‐1‐picrylhydrazyl (DPPH) and 2,2′‐Azino‐bis(3‐ethylbenzothiazoline‐6‐sulfonic acid) (ABTS) radical scavenging methods, demonstrating significant bioactive potential.

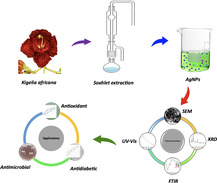

## 1 Introduction

The intriguing domain of nanotechnology explores the advancement and alteration of nanoparticles, which are tiny materials measuring from one nanometer to one hundred nanometers [1, 2]. These particles exhibit significant potential for application across various domains, particularly in health, agriculture, cosmetics, the food sector, oncology treatment, and catalysis [3, 4]. Metal nanoparticles (MNPs) synthesized via sustainable strategies are becoming an attractive topic due to their energy efficiency, safety, cost‐effectiveness, and environmentally friendly nature [5, 6]. The physics, surface interactions, physical, and light‐related properties of MNPs are vastly different compared to their mass counterparts [7]. Due to their unique properties and versatility, AgNPs have enjoyed much attention from researchers of nanomaterials as compared to other MNPs [8, 9, 10]. Silver nanoparticles find use in the preparation of ointments, lotions, and sunscreen products [11, 12, 13]. More than that, AgNPs have demonstrated a high antibacterial activity. Three common methods exist in the synthesis of nanoparticles, namely chemical, physical, and green synthesis [14, 15, 16]. Physical methods of production of the nanomaterials are highly expensive and need special equipment, controlled temperature provision, and pressurization. The use of costly metal salts, stabilizers, reductants, and numerous unsafe solvents in the laboratory manufacture of AgNPs poses serious threats to human health and environmental safety. These shortcomings are accompanied by the limited application of physicochemical techniques as nanoparticle production channels [17, 18, 19]. This, therefore, requires a technique that is quick, easy, cost‐effective, and sustainable. The environmentally friendly method of producing MNPs has garnered notable focus because of its minimal risk. Green production is characterized by its safety, cost‐effectiveness, low toxicity, and reproducibility, making it a viable alternative to various physicochemical processes [20]. Moreover, the process of large‐scale AgNP production is facilitated by environmentally friendly methods. The synthesis of AgNPs derived from different plant components as organic resources has been extensively documented in a multitude of scholarly articles. Examples of the components of plants include leaves, flowers, fruit, roots, and bark. A wide variety of phytoconstituents, including phenolic compounds, proteins, flavonoids, terpenoids, enzymes, and saponins, are present in plant extracts [21]. The compounds found in these plants possess the capability to reduce metallic ions and stabilize nanoparticles to achieve the desired sizes and shapes. Few studies have been published to date regarding the solvent‐based, environmentally friendly production, characterization, and biopotential of AgNPs [22]. The utilization of diverse plant extracts as stabilizing agents, instead of relying on chemicals or intense radiation rays, is referred to as the production of nanoparticles through phytochemical methods [23]. These compounds are significant as they encompass phytochemicals such as polyphenols, in addition to carbohydrates, proteins, and lipids [24]. A burgeoning interdisciplinary field known as “nanomedicine” integrates medical research with nanoscale technologies, resulting in the extensive application of nanomaterials in the medical field. Medicine has evolved beyond the exclusive domain of physicians; nanoscale devices and components are now employed as tools for disease detection, management, and pain relief, in addition to promoting overall health, preservation, and advancement [25]. A variety of pharmacological applications and medicinal strategies utilizing nanoparticles have been granted clinical approval [26]. The application of nanomaterials in the realm of pharmaceuticals, drug delivery, and innovative treatment strategies has demonstrated significant progress in combating various serious diseases [27]. The chemically synthesized approach produces a significant number of nanoparticles rapidly, necessitating the application of sealing agents to maintain the measurements. The materials involved in the creation and stabilization of nanoparticles pose significant risks and generate unwanted by‐products. The growing demand for natural methods of nanoparticle synthesis is primarily driven by the necessity for environmentally friendly chemical approaches in their formation. Green synthesis techniques minimize by‐product formation, enhancing safety and promoting ecological friendliness in the process. Consequently, there is an increasing interest in sustainable nanotechnology solutions. Furthermore, the incorporation of plant products enhances the economic efficiency of synthesizing microbial nanoparticles by reducing expenses associated with bacterial isolation and growth media. Currently, there is a growing interest among individuals in exploring nanobiotechnology within the realm of substance science. Based on their dimensions, dispersion, and shapes, nanostructures can exhibit entirely distinct or enhanced properties [28]. The antibacterial properties of silver‐based compounds are applied in medicine to reduce illness during recovery from thermal injuries and minimally invasive joint surgery, as well as to inhibit bacterial growth on human skin, surgical metals, implants, tubes, cardiac transplants, and dental implants. Owing to their unique attributes, silver nanoparticles are utilized in various fields such as catalytic activity, biochemical discovery, biological sensing, photographic technological advancements, and health care [29]. Silver nanoparticles present numerous possibilities for environmental applications, particularly due to their antibacterial properties. Silver and its nanoparticles find extensive application in health care, particularly in topical lotions designed to mitigate the risk of infections in injuries and untreated cuts. Silver nanoparticles exhibit significant utility in scientific and medical fields due to their attractive physicochemical characteristics. In a recent study by Aouf et al. on biogenic silver nanoparticles of *Moringa oleifera* leaf extraction, characterization and photocatalytic application provide rapid, eco‐friendly approach due to the involvement of active compounds found in plants without the use of toxic chemicals. The crystalline nature and the nanoscale size were determined using X‐ray diffraction (XRD) while zeta potential analysis was used to evaluate surface charge and colloidal stability, the presence of elemental Ag was determined using energy‐dispersive X‐ray analysis, and the shape of the AgNPs was evaluated using field emission scanning electron microscopy and transmission electron microscopy (TEM). *Moringa oleifera* AgNPs exhibited strong photocatalytic activity for degrading MB dye. In particular, the electron–hole pairs formed on the nanoparticle surface highlighted a basic mechanism of photocatalytic activity. Under UV light irradiation for 60 min, biosynthesized AgNPs showed excellent photocatalytic ability with a 98% degradation yield [30].

## 2 Materials and Methods

Silver nitrate (AgNO_3_), Mueller–Hinton agar (MHA), 3‐(4,5‐dimethylthiazol‐2‐yl)−2,5‐diphenyltetrazolium bromide (MTT), 2,2‐diphenyl‐1‐picrylhydrazyl (DPPH), 2,2′‐azinobis(3‐ethylbenzothiazoline‐6‐sulfonic acid) (ABTS), and dimethyl sulfoxide (DMSO) were procured from HiMedia Laboratories. Additional reagents, including sodium hydroxide (NaOH), hydrochloric acid (HCl), phosphate‐buffered saline (PBS), *α*‐amylase, *α*‐glucosidase, *p*‐nitrophenyl‐*α*‐*D*‐glucopyranoside (pNPG), ascorbic acid, gallic acid, Folin–Ciocalteu reagent, methanol, ethanol, and nutrient agar, were employed for nanoparticle synthesis and biological assays. All other supplementary chemicals and solvents used in the study were of analytical grade and obtained from reputable commercial suppliers.

### 2.1 Extraction of Plant Material

#### 2.1.1 Collection of Plant Material and Preparation of Extract

Flowers of the waste *Kigelia africana* plant were collected from the Department of Botany, School of Sciences, IFTM University. Taxonomic identification and authentication of the plant material were carried out at the same institution, and a voucher specimen was deposited under the reference number 2024/SOS/BOT/166. The collected flowers were thoroughly washed with purified water to remove surface impurities and shade‐dried at room temperature. After complete drying, the samples were pulverized into a fine powder using an electric laboratory blender and stored in airtight containers for further experimental use [31].

#### 2.1.2 Extraction

Approximately 100 g of dried flower powder was immersed in 700 mL of ethanol for 5–8 days, with each sample treated separately. The mixture was stirred using a sterilized glass rod every 16 h. The extracts were then filtered using Whatman No. 1 filter paper. The filtrates were concentrated under reduced pressure using a rotary evaporator at 40°C and subsequently stored at 4°C for further use [32].

### 2.2 Phytochemical Analysis

The secondary metabolites present in the waste *K. africana* flower extracts were preliminarily screened to identify phytochemical constituents. Standard qualitative methods, as described by Shukla et al., were employed to detect various compounds, including phenolic constituents [33]. Preliminary qualitative analysis of the phytochemical composition of the plant extract was conducted utilizing established methodologies to ascertain the principal bioactive components. Detection of alkaloids was achieved through the application of Dragendorff's and Mayer's reagents, whereas the presence of flavonoids was validated using the Shinoda test. The evaluation of tannins and phenolic compounds was conducted through the ferric chloride test, which is characterized by the development of a blue–green coloration. Saponins were detected utilizing the foam test, characterized by the sustained formation of froth. Terpenoids were examined through the Salkowski test, which involved the observation of a reddish‐brown interface. The determination of glycosides was conducted utilizing the Keller–Killiani method, while the detection of anthraquinones was achieved through Borntrager's test, which relies on the observation of specific color development. The qualitative assays yielded initial evidence regarding the phytochemical constituents that contribute to biological activity and the stabilization of nanoparticles.

### 2.3 Synthesis of Ka‐AgNPs

To prepare silver nanoparticles sustainably, *K. africana* was used to obtain an ethanolic flower extract to determine the *K. africana* concentration. In this procedure, the 10 ml of the flower extract was added to 90 ml of an aqueous solution of one millimolar concentration of silver nitrate. The blend was stirred as it was cooked at 80°C in 4 h. The color change of the yellow compound to a dark brown color became an indicator that silver nanoparticles (AgNPs) are formed. The nanoparticle was separated through centrifugal action at a speed of 20,000 revolutions per minute for a period of 20 min. This was repeated three times to ensure the elimination of unreacted silver ions. These nanoparticles, henceforth called Ka‐AgNPs, were freeze‐dried and stored at 4°C to be used in the future [34].

### 2.4 Characterization of Ka‐AgNPs

Several analytical methods were used in the characterization of Ka‐AgNPs [35]. UV–Visible spectroscopy (Shimadzu UV‐1800 UV–Vis spectrophotometer) was applied to monitor the NPs formation by the surface plasmon resonance (SPR). Fourier transform infrared spectroscopy (FTIR) (Bruker Alpha II FTIR) was applied to detect the functional groups involved in silver ion reduction and stabilization. The sample characteristics, i.e., the average particle size, as well as the crystalline structure, were determined using XRD (PANalytical X’Pert PRO X‐ray diffractometer) analysis. Scanning electron microscopy (SEM) (Hitachi SU3500 SEM) was used to build information on the shape and surface topography of the produced NPs. TEM (Model: FEI Tecnai G2 20, USA) confirmed particle size and shape.

### 2.5 Antioxidant Activity

#### 2.5.1 DPPH Free Radical Scavenging Assay

The antioxidant capacity of the Ka‐AgNPs was tested with the DPPH free radical scavenging method. In the procedure, ascorbic acid, a standard, and Ka‐AgNPs of various concentrations (10, 20, 50, 75, and 100 g/mL) were added to a 100 L capacity of the DPPH solution (0.1 mM in 80% ethanol). The ratio between DPPH radical scavenging activity and the percentage was computed using a particular formula [36].

$$\%\;\text{of Inhibition}\;=\;\frac{\left(\text{Absorbance of control}-\text{Absorbance of extract}\right)}{\text{Absorbance of control}}\times 100$$

#### 2.5.2 ABTS Free Radical Scavenging Assay

The assessment of the ABTS radical scavenging capacity of Ka‐AgNPs was conducted at various concentrations following a standardized protocol [37]. A stock solution of ABTS was created by combining 7 mM ABTS with 2.45 mM K_2_S_2_O_8_ and then incubating it in the dark at ambient temperature for 20 h. The stock solution was diluted with EtOH to achieve an absorbance of 0.85 ± 0.20 at 734 nm, leading to the creation of the working solution. The solution was permitted to rest at ambient temperature for 35 min, and absorbance was recorded at 734 nm using a Systronic UV‐1800 spectrophotometer.

### 2.6 Antidiabetic Activity

Ka‐AgNPs were also examined for the possibility of preventing diabetes using the *α*‐amylase and *α*‐glucosidase assay.

#### 2.6.1 Inhibition Assay of α‐Amylase

The inhibitory activity in the given test (inhibition of enzyme 1) was conducted with slight modifications according to the approach given by Kaur et al. [38]. Acarbose (AC) inhibitor of *α*‐amylase was used as the reference compound. IC_50_ values of all the enzymes tested, except 1‐1‐*α‐*amylase, were expressed as a percentage of inhibition.

#### 2.6.2 Assessment of α‐Glucosidase Inhibition

The evaluation for *α*‐glucosidase enzyme inhibition was performed with slight adjustments to the protocol described by Kaur et al. [39]. Acarbose served as the reference drug for the inhibition assay, with the absorbance of released *p*‐nitrophenol measured at ≈400 nm. All experiments have been tripled, with the exception of the test substance. The percentage inhibition for enzymes aside from *α*‐glucosidase was assessed and represented as IC_50_ values.

### 2.7 Investigation of Antibacterial Activity through Disk Diffusion Assay

The evaluation of the antibacterial activity of *K* *.* *africana* and the biosynthesized Ka‐AgNPs was conducted using the disk diffusion method [40]. A suspension of the bacterial inoculum was distributed evenly onto solidified MHA plates with the use of a sterile swab. The investigation encompassed bacteria and fungi. Disks were treated with Ka‐AgNPs at concentrations of 20, 50, and 75 μg/mL before being positioned onto the inoculated plates. The plates underwent incubation at 37°C for a duration of 24 h, after which the resulting zones of inhibition were quantified in millimeters

## 3 Results

### 3.1 Phytochemicals

The presence of these phytoconstituents suggests that *K. africana* flower extract possesses significant therapeutic potential. Alkaloids and flavonoids are recognized for their antimicrobial and antioxidant properties [41]. Tannins and phenolic components contribute to astringent and anti‐inflammatory activities. Saponins and glycosides may offer cardioprotective and anti‐inflammatory effects, while terpenoids are often associated with anticancer and analgesic properties. The qualitative phytochemical composition of *K. africana* flower extract is shown in Table 1.

**TABLE 1:** Phytochemicals present in the *K. africana* flower extract.

Phytochemical constituents	Result
Alkaloids	**+**
Flavonoids	**+**
Tannins	**+**
Saponins	**+**
Terpenoids	**+**
Glycosides	**+**
Phenolic compounds	**+**
Anthraquinones	**‐**

### 3.2 UV–Visible Spectroscopy

**FIGURE 1:**
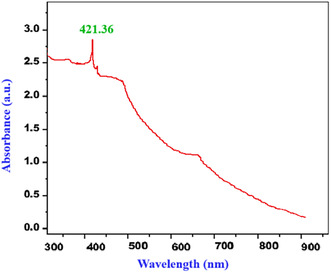
UV–Vis spectrum of phytosynthesized Ka‐AgNPs.

The effective synthesis of Ka‐AgNPs utilizing *K. africana* flower extract was confirmed through a series of spectroscopic analyses. UV–Visible spectrophotometry showed a distinct peak at about 421.36 nm, which is characteristic of AgNPs Figure 1. UV–Vis absorption spectrum of phytosynthesized *K. africana* silver nanoparticles (Ka‐AgNPs). This peak confirmed the reduction of silver ions (Ag^+^) to metallic silver (Ag^0^), facilitated by the phytocompounds found in the flower extract, which likely acted as agents that serve to both reduce and stabilize. The appearance of this peak provides strong evidence for the successful bioreduction of Ag^+^to Ag^0^ by the phytochemicals present in the flower extract. The absence of any peaks at higher wavelengths suggests that the synthesized NPs demonstrated a predominantly spherical shape and relatively small in size, without significant aggregation. The basis for this observation is the SPR principle, which is caused by the collective oscillation of conduction electrons on a nanoparticle's surface in response to light. While nonspherical or anisotropic nanoparticles like rods, triangles, or plates produce additional SPR bands at higher wavelengths due to multiple oscillation modes, spherical nanoparticles usually exhibit a single, sharp SPR peak at a characteristic wavelength (for example, around 420 nm for silver nanoparticles). Thus, it is confirmed that the nanoparticles have a homogeneous and primarily spherical morphology because there is only one clear SPR peak present and no subsidiary peaks at longer wavelengths [42]. The intensity and sharpness of the peak also indicate a relatively narrow size distribution and high stability of the NPs in colloidal form. Moreover, the SPR band position aligns with those reported in earlier studies involving the synthesis of AgNPs using plant extracts, further supporting the efficiency of *K. africana* flower extract as a reducing and stabilizing agent. The two prominent peaks noted at elevated wavelengths are typically ascribed to the existence of biomolecules derived from the plant extract and the potential aggregation of nanoparticles. In the process of green synthesis, a range of phytochemicals, including flavonoids, terpenoids, phenolics, and proteins, function as reducing and stabilizing agents. The absorption of light in the UV and visible regions by these compounds leads to the emergence of additional broad peaks in the spectrum. Additionally, minor broadening or shifting of peaks may arise from variations in particle size distribution, anisotropy, or the partial aggregation of nanoparticles. The small, intense SPR peak indicates the successful formation of silver nanoparticles, whereas the broad peaks reflect the influence of plant metabolites and slight aggregation effects present in the colloidal solution. The UV–Vis data of *K.* flower extract are provided in the Supporting Information (SI) section. The role of compounds having phenolic groups is crucial in this process, as these compounds possess functional groups capable of donating electrons to reduce silver ions while simultaneously stabilizing the nanoparticles. UV–Vis spectral data not only confirm nanoparticle synthesis but also offer insight into particle morphology and stability, validating the environmentally friendly synthesis method utilized in this study. The prominent and stable SPR peak underscores the reliability of *K. africana* flower extract as an eco‐friendly and efficient bioresource for nanoparticle production.

### 3.3 Fourier Transform Infrared Spectroscopy Spectroscopy

FTIR spectroscopy was engaged to determine the functional groups that are found in the *K. africana* flower extract and to understand their involvement in the reduction and stabilization of AgNPs. The FTIR spectrum of the flower before and after nanoparticle synthesis exhibited several key absorption bands corresponding to bioactive compounds known to participate in nanoparticle formation. A broad absorption band observed around 3491.56 cm^−1^ relates to O—H stretching vibrations of hydroxyl groups, typically observed in phenolic compounds and flavonoids. These groups are known for their critical role in the reduction of Ag^+^ to Ag^0^ [43]. Peaks near 2814.24 cm^−1^ are suggestive of C—H stretching vibrations from aliphatic hydrocarbons, indicating the presence of plant‐derived materials and organic compounds.

The intense absorbance at about 1811.32 and 1592.46 cm^−1^ can be attributed to the C=O stretching vibration of carbonyl groups in proteins or flavonoids. The band near ≈1811 cm^−1^ is now attributed to C=O stretching vibrations of anhydrides or esterified carbonyl groups possibly arising from oxidized phytochemicals or organic acids present in the flower extract, while the band at ≈1592 cm^−1^ is reassigned to aromatic C=C stretching and/or asymmetric COO^−^ vibrations associated with phenolic compounds and plant metabolites. These functional groups are known to participate in metal ion reduction and nanoparticle stabilization [44, 45]. This maximum indicates that proteins and polyphenols contained in the flower extract are also implicated in the capping and stabilization of the AgNPs, in addition to being involved in reduction. Further, bands at 1266.23 cm^−1^ (C—N stretching of aromatic amines) and 730.42–1108.22 cm^−1^ (C—O stretching of alcohols or ethers) further indicate the presence of several phytoconstituents actively participating in the synthesis of nanoparticles. The FTIR data of *K. africana* flower extract are provided in the SI section.

**FIGURE 2:**
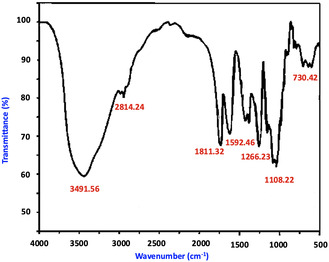
FTIR of phytosynthesized Ka‐AgNPs. FTIR = Fourier transform infrared spectroscopy.

Marginal variations in the intensities and peaks were noticed after the formation of nanoparticles, substantiating an interaction of the functional groups with silver ions during the synthesis process. These changes support the input of hydroxyl, carbonyl, and amine groups to the reduction and stabilization of AgNPs Figure 2. FTIR spectrum of phytosynthesized *K. africana‐*mediated silver nanoparticles (Ka‐AgNPs).

### 3.4 X‐Ray Diffraction Analysis

**FIGURE 3:**
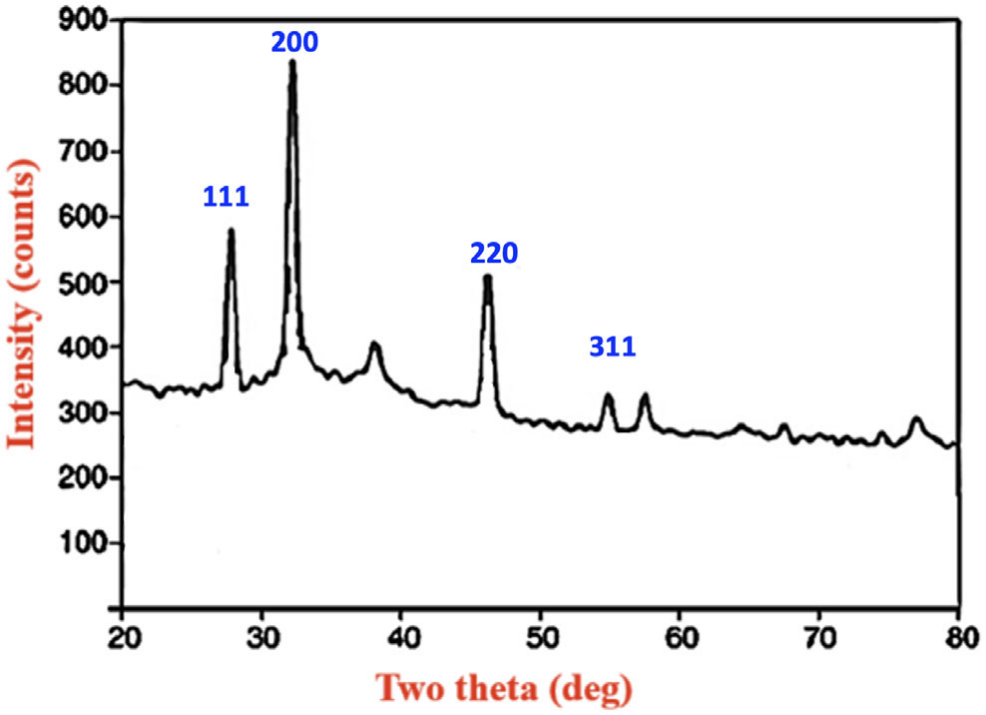
XRD pattern of phytosynthesized Ka‐AgNPs. XRD = X‐ray diffraction.

XRD analysis was performed to investigate the crystalline structure and phase purity of the silver nanoparticles (AgNPs) synthesized using *K. africana* flower extract. The XRD pattern exhibited distinct diffraction peaks at 2*θ* values around 27.1°, 33.3°, 45.5°, and 55.3°**,** which correspond to the (111), (200), (220), and (311) planes of the face‐centered cubic (FCC) crystal structure of Ag, as indexed by the Joint Committee on Powder Diffraction Standards (96‐150‐9854) Table 2. Diffraction peak positions (2*θ*), Miller indices (hkl), full width at half maximum (FWHM), and *d*‐spacing values of *K. africana‐*mediated silver nanoparticles (Ka‐AgNPs). The distinctness and strength of these peaks validate the elevated level of crystallinity of the synthesized nanoparticles. Among them, the (111) plane exhibited the strongest reflection, which is typical for silver nanoparticles and suggests a preferential growth along this plane. The lack of subsequent peaks in the XRD spectrum indicates that the synthesized material is phase‐pure silver without contamination from other crystalline impurities or by‐products [46]. The average crystallite dimensions of the AgNPs were determined utilizing the Debye–Scherrer equation, which typically falls within the range from 20 to 50 nm, aligned with the SEM observations. These results support that the green synthesis route using *K. africana* flower extract not only produces pure silver nanoparticles but also controls their crystalline nature at the nanoscale. The presence of such well‐defined crystalline peaks further indicates that the bioactive compounds in the extract not only diminish the silver ions but additionally impact the nucleation and growth of nanoparticles, promoting orderly crystal formation. This is advantageous for applications where crystallinity significantly impacts performance such as in catalysis, antimicrobial activity, and electronics Figure 3. XRD pattern of phytosynthesized *K. africana‐*mediated silver nanoparticles (Ka‐AgNPs).

**TABLE 2:** Position [°2Th.], hkl, FWHM left, and *d*‐spacing of Ka‐AgNPs.

Pos. (°2Th.)	hkl	FWHM Left (°2Th.)	*d*‐spacing, Å
27.1	111	0.2654	2.33898
33.3	200	0.3865	2.33898
45.5	220	0.4214	2.02961
55.3	311	0.4063	2.02961
Average crystallite size–41.56 nm			

### 3.5 Scanning Electron Microscopy Analysis

**FIGURE 4:**
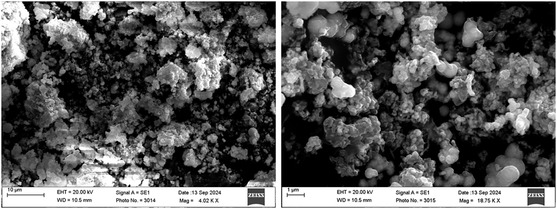
SEM images of the phytosynthesized Ka‐AgNPs. SEM = Scanning electron microscopy.

SEM was conducted to determine the surface morphology, size, and structural features of the AgNPs synthesized using *K. africana* flower extract. The SEM images displayed that the NPs were predominantly spherical to quasispherical in shape with a relatively uniform size distribution. The particle sizes were found to range from ≈60.54 nm, confirming the formation of nanoparticles within the expected nanometer scale [47]. Measurements of particle sizes were conducted utilizing SEM micrographs analyzed through ImageJ. The calibration of the image was performed utilizing the 500 nm scale bar, where 500 nm corresponds to 250 pixels, resulting in a conversion of 2.00 nm per pixel. Thirty distinct particles were measured (excluding agglomerates); the total of the measured diameters was 1816.2 nm, resulting in an average particle diameter of 1816.2/30 = 60.54 nm. The size distribution (mean ± SD, *n* = 30) is detailed in the revised manuscript and SI. The micrographs also showed that the particles were well dispersed, although some moderate agglomeration was observed. This aggregation is a common feature in plant‐mediated synthesis and may result from the drying process during sample preparation or due to natural intermolecular interactions between NPs. However, the presence of smooth surfaces and defined boundaries suggested effective capping and stabilization by the phytochemicals present in the flower extract. The SEM findings correlate well with UV–Vis and XRD results, supporting the establishment of clearly defined, stable, and crystalline AgNPs that the biosynthesis was controlled and efficient, likely due to the existence of flavonoids, phenols, proteins, and other bioactive compounds. SEM analysis confirmed the successful green synthesis of AgNPs with desirable morphological features using *K. africana* flower extract Figure 4. SEM micrographs of phytosynthesized *K. africana‐*mediated silver nanoparticles (Ka‐AgNPs). The dimensions and configuration of the NPs produced via this eco‐friendly route suggest their potential for various biological and environmental applications, including antimicrobial, antioxidant, and catalytic uses.

### 3.6 Transmission Electron Microscopy analysis

**FIGURE 5:**
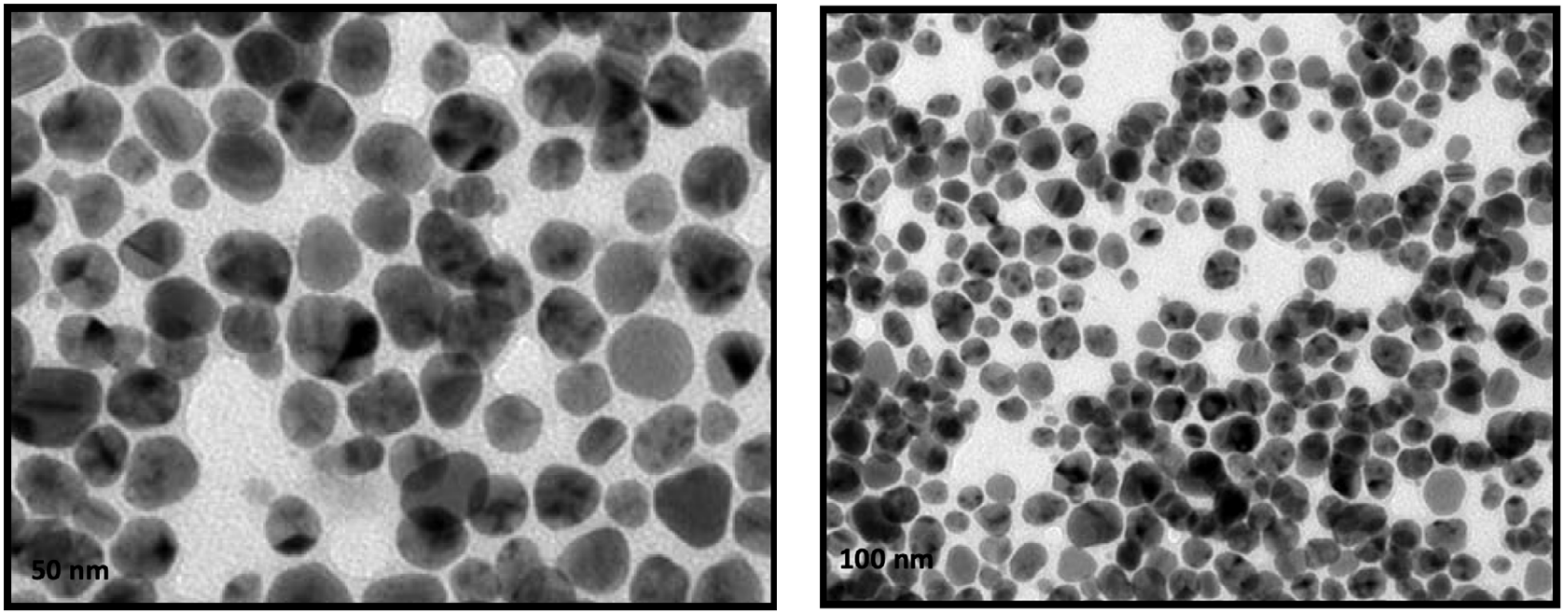
TEM images of the phytosynthesized Ka‐AgNPs. TEM = Transmission electron microscopy.

TEM was used to study the morphology and size distribution of biosynthesized Ka‐AgNPs. The TEM micrographs showed that the AgNPs were mostly spherical with smooth surfaces and well‐defined borders, confirming their effective synthesis Figure 5. TEM micrographs of phytosynthesized *K. africana‐*mediated silver nanoparticles (Ka‐AgNPs). The *K. africana* extract's phytochemicals appeared to cap and stabilize the particles, since they were equally dispersed and did not aggregate. The TEM pictures showed that the synthesized AgNPs ranged in size from 15 to 60 nm, with an average of 35 nm. The limited size distribution suggests a well‐regulated nucleation and growth mechanism during silver ion reduction. TEM pictures show no major agglomerates, indicating that bioactive chemicals and nanoparticle surfaces interact strongly to prevent coalescence. High‐resolution TEM pictures show clear lattice fringes, confirming the crystalline nature of AgNPs and correlating with metallic silver's FCC structure. The results match XRD data, demonstrating that *K. africana* extract reduces and stabilizes green synthesis.

### 3.7 Antioxidant Activity

**FIGURE 6:**
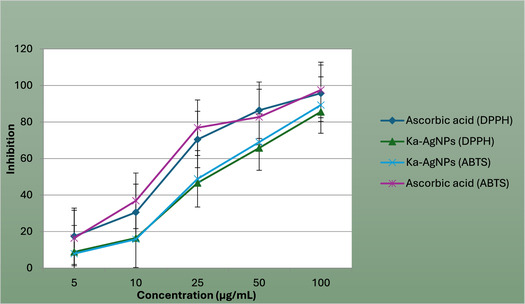
Antioxidant activity by the ABTS and DPPH method of Ka‐AgNPs synthesized from ethanol extract.

The antioxidant potential of *K. africana* flower extract and its green‐synthesized AgNPs was assessed employing DPPH and ABTS radical scavenging analyses. In the DPPH assay, both the extract and AgNPs demonstrated a concentration‐dependent increase in free radical scavenging activity Figure 6. Antioxidant activity of phytosynthesized *Kigelia africana‐*mediated silver nanoparticles (Ka‐AgNPs) evaluated by ABTS and DPPH assays using ethanol flower extract. At a peak concentration of 100 µg/mL, the AgNPs showed an inhibition percentage of 95.6%. The IC_50_ values were found to be 27.88 µg/mL for the AgNPs. These values were compared with the standard antioxidant, ascorbic acid, which demonstrated an IC_50_ of 17.18 µg/mL, indicating that while the standard had the strongest activity, the AgNPs also showed notable efficacy. Similarly, in the ABTS radical scavenging assay, both the extract and AgNPs demonstrated strong antioxidant potential, again in a manner that depends on concentration. At 100 µg/mL, AgNPs inhibited ABTS radicals by 93.3%. The IC_50_ values were 25.27 µg/mL for the AgNPs, compared to 18.95 µg/mL for the ascorbic acid standard. These results indicate that the silver nanoparticles exhibited slightly superior antioxidant potential, likely due to the increased area of surface and the synergistic effect of phytochemicals bound to the nanoparticle surfaces [48].

### 3.8 Antidiabetic Activity

**FIGURE 7:**
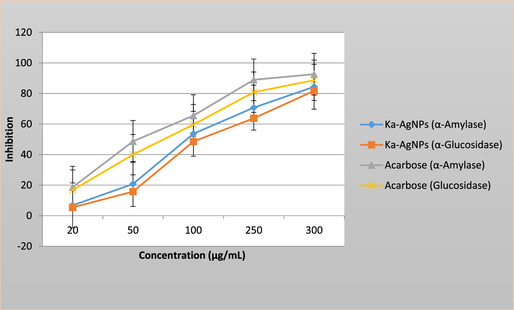
Antidiabetic activity by the *α*‐amylase and *α*‐glucosidase method of Ka‐AgNPs synthesized from ethanol extract.

The antidiabetic activity of *K. africana* flower extract and its biosynthesized silver nanoparticles (AgNPs) was assessed using in vitro inhibition of *α*‐amylase and *α*‐glucosidase enzymes, both of which play key functions in carbohydrate breakdown and postprandial blood glucose regulation Figure 7. In vitro antidiabetic activity of phytosynthesized *K. africana‐*mediated silver nanoparticles (Ka‐AgNPs) evaluated by *α*‐amylase and *α*‐glucosidase inhibition assays using ethanol flower extract. The findings indicated a dose‐dependent inhibitory effect in both assays. In the *α*‐amylase inhibition assay, AgNPs showed a significantly higher inhibition of 81.68% at the 300 µg/mL concentration. The IC_50_ values were established as 98.26 µg/mL. Acarbose, used as the standard inhibitor, illustrated an IC_50_ value of 52.49 µg/mL, indicating that while the standard was more potent, the AgNPs also exhibited promising inhibitory activity. Similarly, in the *α*‐glucosidase inhibition assay, at 250 µg/mL, the AgNPs achieved 80.12% inhibition. The IC_50_ values were 125.34 µg/mL for the AgNPs, compared to 70.17 µg/mL for acarbose. These results indicate that the silver nanoparticles synthesized from *K. africana* possess significant enzyme inhibitory activity. The enhanced activity noticed in the nanoparticle form could be ascribed to the increased surface area, improved bioavailability, and synergistic action of the phytochemicals adsorbed onto the nanoparticle surface [49]. This study provides preliminary in vitro evidence of the antioxidant and *α*‐amylase inhibitory activities of *K. africana‐*derived silver nanoparticles. Further cellular and in vivo studies are required to establish their biological relevance and therapeutic applicability.

### 3.9 Antimicrobial Activity

**FIGURE 8:**
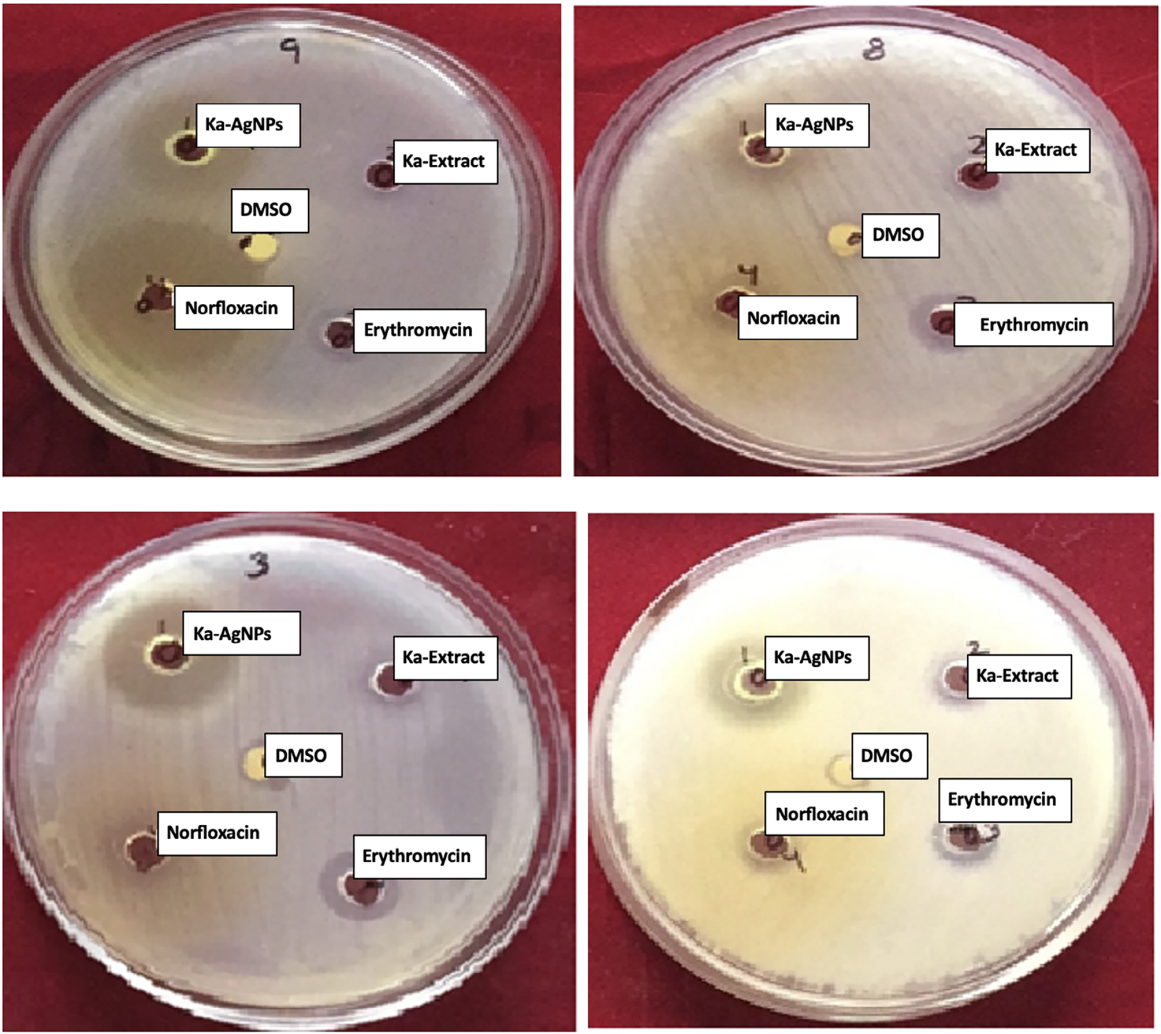
Zone of inhibition by Ka‐AgNPs against the mentioned bacteria at various concentrations.

The biosynthesized AgNPs using *K. africana* flower extract demonstrated significant antimicrobial effectiveness across various pathogenic microorganisms. The antimicrobial efficacy was assessed utilizing the agar well diffusion technique against both Gram‐positive bacteria (*Staphylococcus aureus*, *Bacillus subtilis*) and Gram‐negative bacteria (*Pseudomonas aeruginosa*), as well as fungal strains (*Aspergillus niger*). The zone of inhibition values ranged from 12 to 20 mm, depending on the microorganism and nanoparticle concentration. Notably, *S. aureus* and *P. aeruginosa* exhibited the highest sensitivity, featuring areas of inhibition of 19 and 17 mm, respectively, at 25 µg/mL of AgNPs. The activity was found to be dose dependent, with larger inhibition zones observed at higher concentrations. Compared to the standard antibiotics (norfloxacin and erythromycin), the synthesized nanoparticles showed comparable or even superior antimicrobial effects in some cases [50, 51, 52, 53, 54] Figure 8. Zones of inhibition exhibited by phytosynthesized *K. africana‐*mediated silver nanoparticles (Ka‐AgNPs) against selected bacterial strains at various concentrations. The enhanced activity is ascribed to the small particle size, elevated surface area, and synergistic effect of bioactive phytochemicals from the *K. africana* extract that may stabilize the nanoparticles and contribute to microbial membrane disruption. These findings suggest that *K. africana‐*mediated AgNPs could serve as effective antimicrobial agents for biomedical and environmental applications Table 3. Antimicrobial screening of phytosynthesized *K. africana‐*mediated silver nanoparticles (Ka‐AgNPs, 25 µg/mL) against selected bacterial and fungal strains.

**TABLE 3:** Antimicrobial screening test of Ka‐AgNPs (25 µg/mL) against bacterial and fungal stain.

Bacteria species	Ka‐AgNPs	Ka extract	DMSO	Norfloxacin	Erythromycin
*Staphylococcus aureus*	20 ± 0.12	15 ± 0.20	ND	18 ± 0.23	18 ± 0.03
*Bacillus subtilis*	17 ± 0.05	14 ± 0.14	ND	19 ± 0.10	18 ± 0.10
*Pseudomonas aeruginosa*	12 ± 0.04	11 ± 0.05	ND	18 ± 0.15	17 ± 0.14
*Aspergillus niger*	17 ± 0.01	14 ± 0.3	ND	17 ± 0.11	16 ± 0.12

The comparative study of green‐synthesized silver nanoparticles (AgNP) from different biological sources reveals notable variations in their physicochemical and biological properties. Among the plant‐based methods, *Ocimum sanctum* (Tulsi), *Aloe vera*, and *Azadirachta indica* extracts have been widely used, producing spherical AgNP in the size range from 30 to 80 nm [55, 56]. These nanoparticles exhibited excellent antibacterial activity against common pathogens such as *E. coli* and *S. aureus*, primarily due to the presence of bioactive compounds like flavonoids, terpenoids, and phenolics, which act as natural reducing and stabilizing agents. Similarly, *Lawsonia inermis* (Henna) extract produced 40 nm spherical AgNP that demonstrated both antimicrobial and cytotoxic effects against MCF‐7 breast cancer cells, indicating their potential in biomedical applications [57]. In contrast, seed extracts, such as *Cassia tora*, generated slightly larger nanoparticles (55–65 nm), which displayed selective antibacterial efficacy, particularly against Gram‐positive bacteria, likely due to differences in cell wall permeability [58]. The comparative antibacterial activity of silver nanoparticles synthesized using different plant materials is shown in Table 4.

**TABLE 4:** Comparative study of antibacterial activity of silver nanoparticles synthesized using plant material.

Plant name	Part	Bacteria	Inhibition zone	MIC	References
*Ananas comosus*	Peel	*Escherichia coli*	14.3 ± 0.8	32	[59]
*Ananas comosus*	Peel	*Staphylococcus*	12.8 ± 0.6	64	[60]
*Ananas comosus*	Peel	*Candida albicans*	11.5 ± 0.5	64	[59]
*Allium sativum*	Peel	*Escherichia coli*	18.5 ± 0.7	16	[61]
*Azadirachta indica*	Leaf	*Staphylococcus aureus*	18.2 ± 0.6	16	[62]
*Azadirachta indica*	Leaf	*Escherichia coli*	16.5 ± 0.8	32	[63]
*Azadirachta indica*	Leaf	*Candida albicans*	15.3 ± 0.7	16	[63]
*Camellia sinensis*	Leaf	*Escherichia coli*	17.9 ± 0.7	16	[45]
*Citrus sinensis*	Peel	*Escherichia coli*	16.2 ± 0.8	32	[64]
*Citrus sinensis*	Peel	*Listeria monocytogenes*	15.5± 0.02	NA	[64]
*Citrus sinensis*	Peel	*Staphylococcus aureus*	17.3± 0.01	NA	[64]
*Citrus sinensis*	Peel	Pseudomonas aeruginosa	18.12± 0.22	NA	[64]
*Zingiber officinale*	Rhizome	*Staphylococcus aureus*	15.2 ± 0.9	32	[65]

Microbial‐mediated synthesis generally produced smaller and more uniform nanoparticles with enhanced bioactivity. Fungal species, such as *Talaromyces purpureogenus*, yielded AgNP of 30–60 nm, showing strong antibacterial activity against *Listeria monocytogenes* and *Salmonella typhi*. Bacteria, like *Lactobacillus acidophilus*, produced even smaller AgNP (10–20 nm), exhibiting significant inhibition of multidrug‐resistant strains, coupled with biocompatibility and nonhemolytic behavior. Algal and yeast‐mediated synthesis also demonstrated promising results; *Chlorella vulgaris* and *Saccharomyces cerevisiae* generated AgNP with average sizes of 12.8 and 13–60 nm, respectively, exhibiting both antimicrobial and photocatalytic dye‐degradation activities [66]. The uniqueness of this study is found in the environmentally friendly synthesis of silver nanoparticles utilizing *K. africana* flower extract, accompanied by thorough physicochemical characterization and a detailed assessment of the in vitro biological activities. This report presents a novel application of *K. africana* flowers as a reducing and stabilizing agent in the synthesis of silver nanoparticles (AgNPs), providing a comprehensive analysis of the floral phytochemicals involved, as evidenced by FTIR analysis. Moreover, the research incorporates evaluations of structure, morphology, and biological activity, emphasizing the promise of floral extracts as eco‐friendly nanofactories and presenting initial data on their antioxidant, antibacterial, and enzyme inhibitory characteristics, thus laying the groundwork for subsequent in vivo studies.

## 4 Conclusion

This study successfully produced AgNPs utilizing *K. africana* flower extract as a natural reducing and stabilizing agent. Color change and extensive physicochemical analysis validated AgNP production. The UV–Vis examination confirmed AgNPs production with a peak at about 420–430 nm. The FTIR analysis provided confirmation regarding the participation of flavonoids, phenolic compounds, and proteins in the reduction and stabilization (capping) of the synthesized nanoparticles. XRD showed a FCC structure for the nanoparticles, whereas SEM showed spherical particles with uniform dispersion and nanoscale size distribution. The synthesized AgNPs showed substantial free radical scavenging in DPPH and ABTS tests, suggesting that they might neutralize oxidative stress. The NPs also exhibited dose‐dependent antidiabetic effects, suggesting that they could be used to treat postprandial hyperglycemia. Like antibiotics, AgNPs showed broad‐spectrum antibacterial efficacy against Gram‐positive and Gram‐negative bacteria, as well as fungal strains, with large inhibition zones. This study demonstrates that *K. africana‐*mediated AgNPs can be utilized in antioxidant therapy, diabetic management, and antibacterial defense through eco‐friendly synthesis.

## Supporting Information

Additional SI can be found online in the Supporting Information section. **Supporting Fig. S1**: UV–Vis spectrum of ethanolic extract of *Kigelia africana* flower. **Supporting Fig. S2**: FT‐IR of ethanolic extract of *Kigelia africana* flower. **Supporting Fig. S3**: Authentication letter of *Kigelia africana* flower.

## Conflicts of Interest

The authors declare no conflicts of interest.

## Supporting information

Supplementary Material

## Data Availability

The data that support the findings of this study are available from the corresponding author upon reasonable request.

## References

[1] S. M. Saleh , M. K. Almotiri , and R. Ali , “Green synthesis of highly luminescent gold nanoclusters and their application in sensing Cu(II) and Hg(II),” Journal of Photochemistry and Photobiology A: Chemistry 426 (2022): 113719.

[2] R. Ali , B. Alfeneekh , S. Chigurupati , and S. M. Saleh , “Green Synthesis of Pregabalin‐Stabilized Gold Nanoclusters and Their Applications in Sensing and Drug Release,” Archives of Pharmacy 355, no. 4 (2022): 2100426. 10.1002/ardp.202100426 35088474

[3] A. A. Kadhim , N. R. Abbas , H. H. Kadhum , et al., “Investigating the Effects of Biogenic Zinc Oxide Nanoparticles Produced Using Papaver Somniferum Extract on Oxidative Stress, Cytotoxicity, and the Induction of Apoptosis in the THP‐1 Cell Line,” Biological Trace Element Research 201, no. 10 (2023): 4697–4709. 36662347 10.1007/s12011-023-03574-7

[4] R. J. B. Peters , H. Bouwmeester , S. Gottardo , et al., “Nano Materials for Products and Application in Agriculture, Feed and Food,” Trends in Food Science and Technology 54 (2016): 155–164.

[5] G. Fytianos , A. Rahdar , and G. Z. Kyzas , “Nanomaterials in Cosmetics: Recent Updates,” Nanomaterials 10, no. 5 (2020): 979. 32443655 10.3390/nano10050979 PMC7279536

[6] A. Kaur , H. Gupta , and S. Dhiman , “Biogenic Metallic Nanoparticles: Synthesis and Applications Using Medicinal Plants,” in Handbook of Green and Sustainable Nanotechnology: Fundamentals, Developments and Applications (Springer International Publishing), 1–23.

[7] S. M. Saleh , F. M. Alminderej , R. Ali , and O. I. Abdallah , “Optical Sensor Flm for Metribuzin Pesticide Detection,” Spectrochimica Acta Part A: Molecular and Biomolecular Spectroscopy 229 (2020): 117971. 31954291 10.1016/j.saa.2019.117971

[8] S. M. Saleh , R. Ali , M. E. F. Hegazy , F. M. Alminderej , and T. A. Mohamed , “Te Natural Compound Chrysosplenol‐D Is a Novel, Ultrasensitive Optical Sensor for Detection of Cu(II),” Journal of Molecular Liquids 302 (2020): 112558.

[9] S. M. Saleh , W. A. El‐Sayed , M. A. El‐Manawaty , M. Gassoumi , and R. Ali , “Aneco‐Friendly Synthetic Approach for Copper Nanoclusters and Their Potential in Lead Ions Sensing and Biological Applications,” Biosensors 12, no. 4 (2022): 197. 35448257 10.3390/bios12040197 PMC9032517

[10] S. M. Saleh , “ZnO Nanospheres Based Simple Hydrothermal Route for Photocatalytic Degradation of Azo Dye,” Spectrochimica Acta Part A: Molecular and Biomolecular Spectroscopy 211 (2019): 141–147. 30530067 10.1016/j.saa.2018.11.065

[11] M. Tayyab , Y. Liu , Z. Liu , et al., “One‐Pot in‐Situ Hydrothermal Synthesis of Ternary In_2_S_3_/Nb_2_O_5_/Nb_2_C Schottky/S‐Scheme Integrated Heterojunction for Efficient Photocatalytic Hydro Gen Production,” Journal of Colloid and Interface Science 628 (2022): 500–512. 36007415 10.1016/j.jcis.2022.08.071

[12] E. Albadri , M. A. B. Aissa , A. Modwi , and S. M. Saleh , “Synthesis of Mesoporous Ru‐ZnO g‐C_3_N_4_ Nanoparticles and Their Photocatalytic Activity for Methylene Blue Degradation,” Water 15, no. 3 (2023): 481.

[13] S. M. Saleh , A. E. Albadri , M. A. B. Aissa , and A. Modwi , “Fabrication of Mesoporous V_2_O_5_@ g‐C_3_N_4_ Nanocomposite as Photocatalyst for Dye Degradation,” Crystals 12 (2022): 1766.

[14] W. Abdussalam‐Mohammed , “Comparison of Chemical and Biological Properties of Metal Nanoparticles (Au, Ag), with Metal Oxide Nanoparticles (ZnO‐NPs) and Their Applications,” Advanced Journal of Chemistry, Section A 3 (2020): 192–210.

[15] H. M. Fahmy , A. M. Mosleh , A. A. Elghany , et al., “Coated Silver Nanoparticles: Synthesis, Cytotoxicity, and Optical Properties,” RSC Advances 9, no. 35 (2019): 20118–20136. 35514687 10.1039/c9ra02907a PMC9065456

[16] K. P. Steckiewicz , P. Cieciórski , E. Barcińska , et al., “Silver Nanoparticles as Chlorhexidine and Metronidazole Drug De Livery Platforms: Their Potential use in Treating Periodontitis,” International Journal of Nanomedicine 17 (2022): 495–517. 35140461 10.2147/IJN.S339046 PMC8820264

[17] J. M. Domingues , C. S. Miranda , N. C. Homem , H. P. Felgueiras , and J. C. Antunes , “Nanoparticle Synthesis and Their Integration into Polymer‐Based Fibers for Biomedical Applications,” Biomedicines 11, no. 7 (1862): 2023. 10.3390/biomedicines11071862 PMC10377033 37509502

[18] S. Gupta , M. V. Singh , M. Rani , S. Arora , S. D. Sharma , and A. Kaur , “Transforming Fruit and Vegetable Waste into Nanoparticles: A Step Towards Sustainable Nanotechnology,” Natural Product Communications 20, no. 6 (2025): 1934578X251346010.

[19] P. Dikshit , J. Kumar , A. Das , et al., “Green Synthesis of Metallic Nanoparticles: Applications and Limitations,” Catalysts 11, no. 8 (2021): 902.

[20] A. Singh , B. L. R. Madhavi , and M. N. Nithin Sagar , “An Overview of Green Synthesis Mediated Metal Nanoparticles Preparation and Its Scale up Opportunities,” Journal of Drug Delivery and Therapeutics 11, no. 6 (2021): 304–314.

[21] A. Zuhrotun , D. J. Oktaviani , and A. N. Hasanah , “Bio Synthesis of Gold And Silver Nanoparticles Using Phytochemical Compounds,” Molecules 28, no. 7 (2023): 3240–3331. 37050004 10.3390/molecules28073240 PMC10096681

[22] Vidyasagar , R. R. Patel , S. K. Singh , and M. Singh , “Green Synthesis of Silver Nanoparticles: Methods, Biological Applications, Delivery and Toxicity,” Materials Advances 4, no. 8 (2023): 1831–1849.

[23] A. Malakar , S. R. Kanel , C. Ray , D. D. Snow , and M. N. Nadagouda , “Nanomaterials in the Environment, Hu Man Exposure Pathway, and Health Effects: A Review,” Science of the Total Environment 759 (2021): 143470. 33248790 10.1016/j.scitotenv.2020.143470

[24] S. Mohanaparameswari , M. Balachandramohan , P. Sasikumar , et al., “Investigation of Structural Properties and Antibacterial Activity of AgO Nanoparticle Extract from Solanum Nigrum/Mentha Leaf Extracts by Green Synthesis Method,” Green Processing and Synthesis 12, no. 1 (2023): 20230080.

[25] A. Rónavári , N. Igaz , D. I. Adamecz , et al., “Green Silver and Gold Nanoparticles: Biological Synthesis Approaches and Potentials for Biomedical Applications,” Molecules 26, no. 4 (2021): 844. 33562781 10.3390/molecules26040844 PMC7915205

[26] S. H. Lee and B. H. Jun , “Silver Nanoparticles: Synthesis and Application for Nanomedicine,” International Journal of Molecular Sciences 20, no. 4 (2019): 865, 10.3390/IJMS20040865. 30781560 PMC6412188

[27] A. Tuomela , “Nanocrystals for Drug Delivery Applications,” Helsinki 5, no. 1 (2015): 1–6.

[28] S. Iravani , H. Korbekandi , S. V. Mirmohammadi , and B. Zolfaghari , “Synthesis of Silver Nanoparticles: Chemical, Physical and Biological Methods,” Research in Pharmaceutical Sciences 9, no. 6 (2014): 385. 26339255 PMC4326978

[29] N. Krithiga , A. Rajalakshmi , and A. Jayachitra , “Green Synthesis of Silver Nanoparticles Using Leaf Extracts of Clitoriaternatea and Solanum Nigrum and Study of Its Antibacterial Effect against Common Nosocomial Pathogens,” Journal of Nanoscience 2015 (2015): 1–8, 10.1155/2015/928204.

[30] D. Aouf , Y. Khane , F. Fenniche , et al., “Biogenic Silver Nanoparticles of Moringa Oleifera Leaf Extract: Characterization and Photocatalytic Application,” Nanotechnology Reviews 13, no. 1 (2024): 20240002.

[31] E. O. Mikhailova , “Silver Nanoparticles: Mechanism of Action and Probable Bio‐Application,” Journal of Functional Biomaterials 11, no. 4 (2020): 84, 10.3390/JFB11040084. 33255874 PMC7711612

[32] A. Kaur , M. V. Singh , N. Bhatt , S. Arora , and A. Shukla , “Exploration of Chemical Composition and Biological Activities of the Essential Oil from Ehretia acuminata R. Br. Fruit,” ES Food & Agroforestry 15 (2023): 1068.

[33] A. Shukla , P. Pokhriyal , R. K. Shukla , and A. Kaur , “A Green and Facile Approach For Antidiabetic and Anti‐Inflammatory Potency for Ficus Subincisa Fruit,” Indian Drugs 58, no. 08 (2021): 68–74.

[34] P. Pokhriyal , A. Kaur , A. Shukla , S. Dhiman , and H. Gupta , “Biocompatible Zinc Nanoparticles Synthesis from Ficussubincisa for a Sustainable Tomorrow: Characterization and Therapeutic Applications,” Russian Journal of Bioorganic Chemistry 50, no. 2 (2024): 408–417.

[35] A. Kaur , A. Shukla , and R. K. Shukla , “In Vitro Antidiabetic and Anti‐Inflammatory Activities of the Bark of Ehretia Acuminata R. Br,” Indian Journal of Natural Products and Resources (IJNPR)[Formerly Natural Product Radiance (NPR)] 12, no. 4 (2022): 538–543.

[36] I. Khan , K. Saeed , and I. Khan , “Nanoparticles: Properties, Applications and Toxicities,” Arabian Journal of Chemistry 12, no. 7 (2019): 908–931, 10.1016/J.ARABJC.2017.05.011.

[37] A. Kaur , M. V. Singh , A. Shukla , et al., “Isolation, Characterization, and Utilization of Secondary Metabolites from Ehretia Acuminata: Bioactive Compounds for Pharmaceutical and Industrial Applications,” ES Food & Agroforestry 20 (2025): 1447.

[38] A. Shukla , A. Kaur , and R. K. Shukla , “Evaluation of Different Biological Activities of Leaves of, *Ehretia acuminata* R. Br,” Indian Drugs 58, no. 4 (2021): 42–49.

[39] T. Gunasekaran , T. Nigusse , and M. D. Dhanaraju , “Silver Nanoparticles as Real Topical Bullets for Wound Healing,” Journal of the American College of Clinical Wound Specialists 3, no. 4 (2011): 82, 10.1016/J.JCWS.2012.05.001. 24527370 PMC3921230

[40] S. A. Adebayo , J. P. Dzoyem , L. J. Shai , and J. N. Eloff , “The Anti‐Inflammatory and Antioxidant Activity of 25 Plant Species Used Traditionally to Treat Pain in Southern African,” BMC Complementary and Alternative Medicine 15 (2015): 159. 26014115 10.1186/s12906-015-0669-5 PMC4443658

[41] C. P. Khare , Indian Medicinal Plants: An Illustrated Dictionary (Springer‐Verlag, 2007), 205–206.

[42] G. Habibullah , J. Viktorova , and T. Ruml , “Current Strategies for Noble Metal Nanoparticle Synthesis,” Nanoscale Research Letters 16, no. 1 (2021): 47. 33721118 10.1186/s11671-021-03480-8 PMC7960878

[43] E. A. Odongo , P. C. Mutai , B. K. Amugune , N. N. Mungai , M. O. Akinyi , and J. Kimondo , “Evaluation of the Antibacterial Activity of Selected Kenyan Medicinal Plant Extract Combinations against Clinically Important Bacteria,” BMC Complementary Medicine and Therapies 23, no. 1 (2023): 100. 37013533 10.1186/s12906-023-03939-4 PMC10069043

[44] E. Galata , C. M. Veziri , G. V. Theodorakopoulos , G. E. Romanos , and E. A. Pavlatou , “Composite GO/Ceramic Membranes Prepared via Chemical Attachment: Characterisation and Gas Permeance Properties,“ Membranes 12, no. 12 (2022): 1181. 36557088 10.3390/membranes12121181 PMC9787500

[45] S. Ahmed , Saifullah , M. Ahmad , B. L. Swami , and S. Ikram , “Green Synthesis of Silver Nanoparticles Using Azadirachta Indica Aqueous Leaf Extract,” Journal of Radiation Research and Applied Sciences 9, no. 1 (2016): 1–7.

[46] K. Mohlala , U. Offor , E. Monageng , N. B. Takalani , and C. S. Opuwari , “Overview of the Effects of Moringa Oleifera Leaf Extract on Oxidative Stress and Male Infertility: A Review,” Applied Sciences 13, no. 7 (2023): 4387.

[47] J. Tchekalarova and R. Tzoneva , “Oxidative Stress and Aging as Risk Factors for Alzheimer's Disease and Parkinson's Disease: The Role of the Antioxidant Melatonin,” International Journal of Molecular Sciences 24, no. 3 (2023): 3022. 36769340 10.3390/ijms24033022 PMC9917989

[48] H. Vergara‐Castañeda , L. O. Granados‐Segura , G. Luna‐Bárcenas , et al., “Gold Nanoparticles Bioreduced by Natural Extracts of Arantho (Kalanchoe Daigremontiana) for Biological Purposes: Physicochemical, Antioxidant and Antiproliferative Evaluations,” Materials Research Express 6, no. 5 (2019): 055010.

[49] B. Zamani , F. Taghvaee , H. Akbari , A. Mohtashamian , and N. Sharifi , “Effects of Selenium Supplementation on the Indices of Disease Activity, Inflammation and Oxidative Stress in Patients with Rheumatoid Arthritis: A Randomized Clinical Trial,” Biological Trace Element Research 202, no. 4 (2024): 1457–1467. 37477848 10.1007/s12011-023-03782-1

[50] N. Bhatt and M. S. Mehata , “A Sustainable Approach to Develop Gold Nanoparticles with Kalanchoe Fedtschenkoi and Their Interaction with Protein and Dye: Sensing and Catalytic Probe,” Plasmonics 18, no. 3 (2023): 845–858.

[51] R. Lozano‐Rosas , J. J. Ruíz‐Osorio , R. Ramos‐García , R. Silva‐González , T. Spezzia‐Mazzocco , and M. D. J. Robles‐Águila , “Photoexcitation of Ag‐Doped TiO_2_ Nanoparticles with Visible Light for Antimicrobial Photodynamic Therapy against Candida Albicans,” Journal of Nanoparticle Research 27 (2025): 236, 10.1007/s11051-025-06432-w.

[52] N. Bhimani , U. Rathod , A. Ravalia , et al., “Multifunctional CuO/NiO Nanocomposites: A Study of Structural, Spectroscopic, Antibacterial and Antioxidant Properties,” Journal of Nanoparticle Research 27 (2025): 232, 10.1007/s11051-025-06433-9.

[53] A. Verma , P. Dhiman , C. W. Lai , et al., “Fabrication of Visible Light‐Driven Magnetically Separable Ag_3_PO_4_/Fe_3_O_4_/g‐C_3_N_4_ Photo Catalyst for Tetracycline Degradation,” Journal of Nanoparticle Research 27 (2025): 210, 10.1007/s11051-025-06392-1.

[54] Z. Nkentsha and S. Rambharose , “Green‐Synthesized Gold Nanoparticles Exhibit Neuroprotective Activity against Oxidative Stress‐Induced Damage in SH‐SY5Y Cells,” Journal of Nanoparticle Research 27 (2025): 197, 10.1007/s11051-025-06387-y.

[55] Y. L. Tiki , L. D. Tolesa , A. H. Tiwikrama , and T. F. Chala , “Ginger (*Zingiber officinale*)‐Mediated Green Synthesis of Silver‐Doped Tin Oxide Nanoparticles and Evaluation of Its Antimicrobial Activity,” ACS Omega 9, no. 10 (2024): 11443–11452. 38496979 10.1021/acsomega.3c07855 PMC10938312

[56] N. N. Hussein , K. Al‐Azawi , G. M. Sulaiman , et al., “Silver‐Cored Ziziphus Spina‐Christi Extract‐Loaded Antimicrobial Nanosuspension: Overcoming Multidrug Resistance,” Nanomedicine 18, no. 25 (2023): 1839–1854. 37982771 10.2217/nnm-2023-0185

[57] S. A. Kumari , A. K. Patlolla , and P. Madhusudhanachary , “Biosynthesis of Silver Nanoparticles Using Azadirachta Indica and Their Antioxidant and Anticancer Effects in Cell Lines,” Micromachines 13, no. 9 (2022): 1416. 36144039 10.3390/mi13091416 PMC9506441

[58] E. Alhomaidi , S. A. Jasim , H. I. Amin , et al., “Biosynthesis of Silver Nanoparticles Using Lawsonia Inermis and Their Biomedical Application,” IET Nanobiotechnology 16, no. 7‐8 (2022): 284–294. 36039655 10.1049/nbt2.12096 PMC9469786

[59] A. Rahman , N. Jamil , M. Yasir , et al., “Toxicity of Nanomaterials in the Environment: A Critical Review of Current Understanding and Future Directions,” Journal of Nanoparticle Research 27 (2025): 147, 10.1007/s11051-025-06345-8.

[60] A. Baran , C. Keskin , M. F.. Baran , et al., “Ecofriendly Synthesis of Silver Nanoparticles Using Ananas Comosus Fruit Peels: Anticancer and Antimicrobial Activities,” Bioinorganic Chemistry and Applications 2021, no. 1 (2021): 2058149. 34887909 10.1155/2021/2058149 PMC8651404

[61] E. E. Emeka , O. C. Ojiefoh , C. Aleruchi , et al., “Evaluation of Antibacterial Activities of Silver Nanoparticles Green‐Synthesized Using Pineapple Leaf (*Ananas comosus)* ,” Micron 57 (2014): 1–5. 24268599 10.1016/j.micron.2013.09.003

[62] T. Gabriel , A. Vestine , K. D. Kim , S. J. Kwon , I. Sivanesan , and S. C. Chun , “Antibacterial Activity of Nanoparticles of Garlic (Allium Sativum) Extract against Different Bacteria Such as Streptococcus Mutans and Poryphormonas Gingivalis,” Applied Sciences 12, no. 7 (2022): 3491.

[63] A. A. Pawar , J. Sahoo , A. Verma , et al., “ *Azadirachta indica* ‐Derived Silver Nanoparticle Synthesis and Its Antimicrobial Applications,” Journal of Nanomaterials 2022, no. 1 (2022): 4251229.

[64] K. Tran Khac , H. Hoang Phu , H. Tran Thi , V. Dinh Thuy , and H. Do Thi , “Biosynthesis of Silver Nanoparticles Using Tea Leaf Extract (*Camellia sinensis*) for Photocatalyst and Antibacterial Effect,” Heliyon 9, no. 10 (2023): e20707. 37860560 10.1016/j.heliyon.2023.e20707 PMC10582344

[65] A. Hatipoğlu , A. Baran , C. Keskin , et al., “Green Synthesis of Silver Nanoparticles Based on the Raphanus Sativus Leaf Aqueous Extract and Their Toxicological/Microbiological Activities,” Environmental Science and Pollution Research 24 (2023): 1–3. 10.1007/s11356-023-26499-z 36964465

[66] M. S. Nawabjohn , P. Sivaprakasam , S. K. Anandasadagopan , A. A. Begum , and A. K. Pandurangan , “Green Synthesis and Characterisation of Silver Nanoparticles Using Cassia Tora Seed Extract and Investigation of Antibacterial Potential,” Applied Biochemistry and Biotechnology 194, no. 1 (2022): 464–478. 34611854 10.1007/s12010-021-03651-4

