# Supplementary material for: Phytosynthesized Silver Nanoparticles from Waste Kigelia africana Flowers: Characterization and Functional Applications
Source: ChemistryOpen. 2026 Mar 26;15(4):e70187. doi: 10.1002/open.70187 (PMC13140520; doi:10.1002/open.70187)
Supplement: Supplementary file 1 — Supplementary Material [file OPEN-15-e70187-s001.pdf]

Supporting Information

Phyto-synthesized Silver Nanoparticles from Waste *Kigelia africana* Flowers: Characterization and Functional Applications

Lakshya[1a], Amanpreet Kaur[2*], Utkarsh Tyagi[1b], Man Vir Singh[3*], Aaysha Pandey[4], Kamal Kishore[5*], Ranjeet Brajpuria[6], Soniya Dhiman[7], Naresh Kumar Wagri[8]

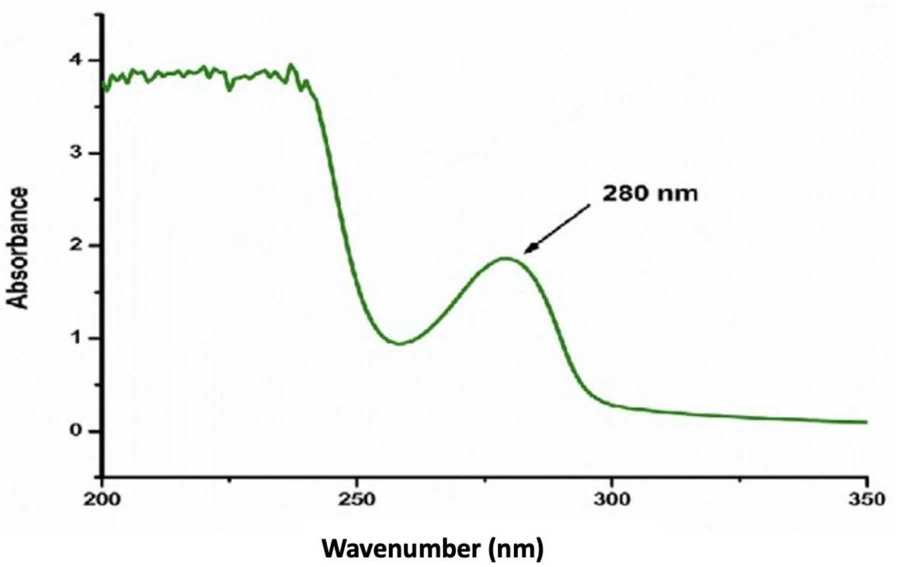

Figure 1. UV–Vis spectrum of ethanolic extract of *Kigelia africana* flower

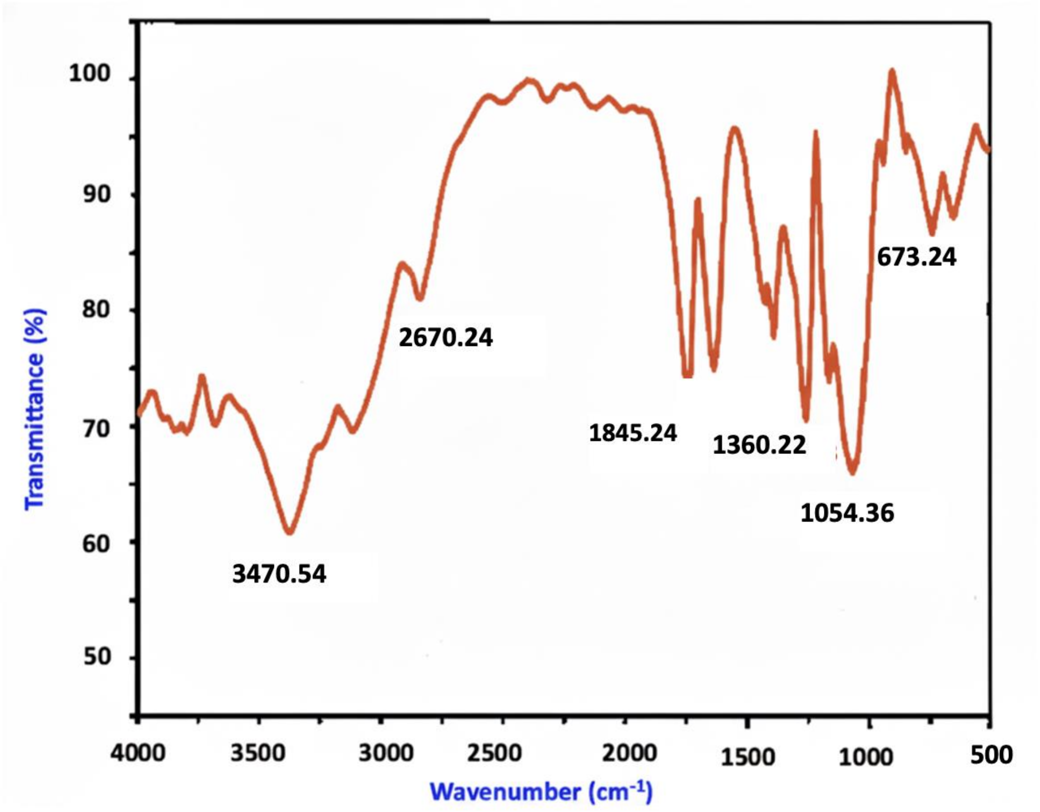

Figure 2. FT-IR of ethanolic extract of *Kigelia africana* flower

Dr. Ashok Kumar
(Assistant Professor)
Department of Botany, School of Sciences
IFTM University, Moradabad
Email: drakatya81@gmail.com
ashokkumar@iftmuniversity.ac.in
Contact No.: 8279818760

IFTM UNIVERSITY
MORADABAD
NACC Accredited with 'A' Grade

Ref. No. 2024/SOS/BOT/166

Date: May 30, 2024

Certificate of Plant Authentication

It is to certify that on the basis of morphological characters, the plant specimens were authenticated as under and the same are deposited in the Department of Botany, School of Sciences, IFTM University, Moradabad.

S. No.	Botanical Name	Family
1	*Delonix regia*	Fabaceae
2	*Kigelia africana*	Bignoniaceae

(Dr. Ashok Kumar)

To,
Mr. Lakshya
Ph. D. Scholar
School of Biotechnology
IFTM University, Moradabad

Figure:3 Authentication letter of *Kigelia africana* flower
